# Comparison of leg dynamic models for quadrupedal robots with compliant backbone

**DOI:** 10.1038/s41598-022-18536-7

**Published:** 2022-08-26

**Authors:** E. A. Parra Ricaurte, J. Pareja, S. Dominguez, C. Rossi

**Affiliations:** Centre for Automation and Robotics UPM-CSIC, Universidad Politécnica de Madrid, Madrid, Spain

**Keywords:** Electrical and electronic engineering, Mechanical engineering, Biomechanics

## Abstract

Many quadrupeds are capable of power efficient gaits, especially trot and gallop, thanks to their flexible trunk. The oscillations of the system that includes the backbone, the tendons and musculature, store and release elastic energy, helping a smooth deceleration and a fast acceleration of the hindquarters and forequarters, which improves the dynamics of running and its energy efficiency. Forelegs and hindlegs play a key role in generating the bending moment in the trunk. In this paper we present our studies aimed at modeling and reproducing such phenomena for efficient quadrupedal robot locomotion. We propose a model, called mass-mass-spring model, that overcomes the limitation of existing models, and demonstrate that it allows studying how the masses of the legs generate a flexing force that helps the natural bending of the trunk during gallop. We apply our model to six animals, that adopt two different galloping patterns (called *transverse *and *rotatory*), and compare their energy efficiency.

## Introduction

Bio-inspiration and bio-mimetics are growing research fields. In the last years, the animal kingdom has served as an example in the development of several bio-inspired structures and mechanisms, especially in robotics. An example of this is legged locomotion, that allows agile movements, great stability on different terrains, high speed and energy efficient running^1–3^.

Some of the first walking machines were ODEX one^4^, PVII Quadruped Vehicle^5^ and SILO4^6^, that achieved optimal gait patterns through a statically stable walking. These prototypes had important movement restrictions, since at least three of their legs had to be in stance with the ground.

In the field of running legged robots representative examples are KOLT^7^, Scout II^8^, BigDog^9^, Star1ETH^10^ and the MIT Cheetah 2^11^. However, these prototypes do not take advantage of the flexibility of the trunk in order to achieve fast galloping speed and power efficiently as their natural counterparts do, like, e.g., cheetahs, horses and greyhounds^1^.

In our current work, we focus on such key feature, and aim at designing legged robots with high energy efficiency and speed using a compliant backbone. In the literature, research devoted to understanding and developing the mechanics of bending of the trunk can be found. Three types of flexible trunks have been proposed: actuated, semi-actuated and passive.

In the class of robots with actuated trunk, the work presented in^12^ showed how the performance of a quadrupedal can be improved actuated joints that allow flexion and extension of the trunk, compared to a rigid bodied robot. The quadruped robot presented in^13^ was equipped with a tensegrity-based spine that helped maintaining movement and balance during gaits. Lastly, robot Stoch2^14^ showed the advantages of actuated spine.

As far as legged robots with semi-actuated trunk is concerned, a comparison between three types of flexible spines in the Lynx-robot was performed in^15^. The first of such spines was actuated for both flexion and extension movements, while the other two, made of a glass fiber rod with different stiffness, were actuated only for the flexion movement and the extension movement was passive.

Concerning legged robots with passive trunk, in^16^ the effect of trunk flexibility on the dynamics of a quadruped robot running with a bounding gait was studied. This model was composed of two rigid bodies representing the hindquarters and forequarters, connected through a torsion spring. It was demonstrated that at a constant energy level, the trunk oscillation range and the average forward speed are inversely related. In^17^ a robot with a flexible backbone whose stiffness could be changed varying the pressure of pneumatic actuators was used to study the stability of gait pattern for different the trunk stiffnesses. Finally, in^18^ the difference between rigid and passive articulated trunk was investigated. The authors showed that the articulated trunk allowed longer strides and significantly affects the dynamics of the robot as well as its power efficiency. Such effect has been observed also in^19,20^ and^21^ for hopping robots with compliant legs, and on flexible backbones for fish-like robots^22^. Also, a similar effect is also present in insects and birds, whose thoraxes contain compliant structures that accumulate and release energy during the flapping cycle at the benefit of consumption, and also flight stability, see, e.g.^23,24^.

In our current work we are investigating flexible backbones for legged robots, and how this can be exploited for fast and energy efficient running for quadruped robots. We have shown how a flexible spine can greatly help to achieve low power consumption storing and releasing energy during gait. Additionally, a dramatic energy saving can be obtained when the oscillations of the trunk reach a quasi-resonant regime^25^.

It is important to highlight that in most rigid-bodied running quadrupedal robots the legs mass is considered negligible for the purpose of studying robots body dynamics. Such assumption allows a simpler dynamic modeling, and light legs allow faster movements, and therefore faster running. However, when it comes to flexible trunk, this plays a key role, since the motion of the masses of the legs (plus tail and head) and their contact with the ground generates the trunk bending^26,27^.

**Figure 1:**
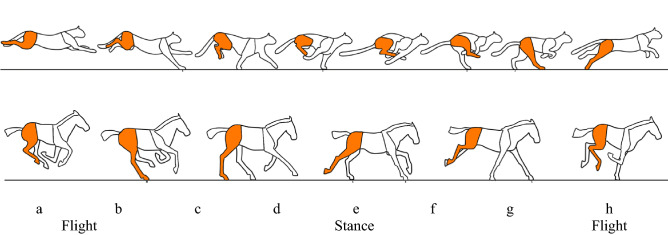
Illustrations of the *stance* and *flight* stages of the rotary gallop of the cheetah (top) and the transverse gallop of the horse (bottom). Adapted from ^26^.

In a running gait, the center of mass of the leg reaches its lowest point at the middle of step. The kinetic energy and gravitational potential energy reaction force are stored as elastic energy during the *stance phase*, when the leg touches the ground, and recovered during the *flight phase*, when the leg leaves the ground (see Fig. 1, steps h, a, b).

**Figure 2:**
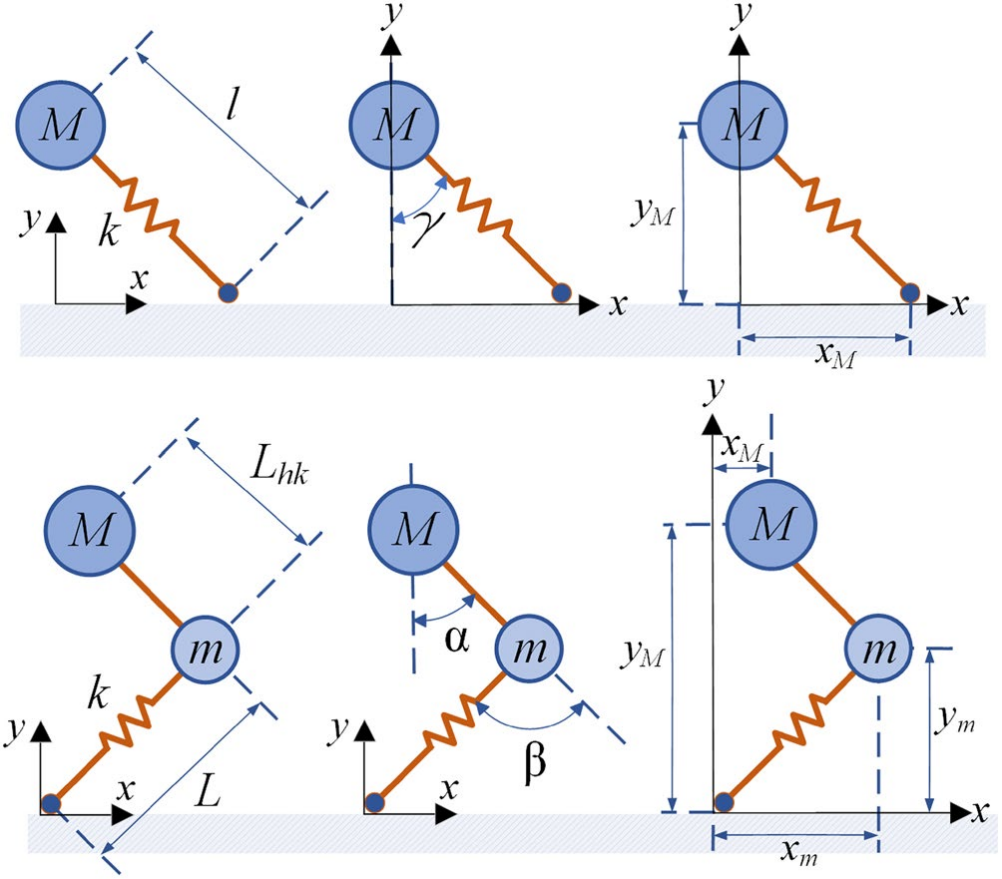
Diagram of the SLIP leg model (top) and Mass-Mass-Spring (MMS) leg model (bottom).

The most studied model for robotic legs for running gaits is the SLIP (Spring Loaded Inverted Pendulum) model (see Fig. 2, top). In the flight phase the spring has no effect and thus is not considered, so the dynamics of the leg is represented taking into account only the point mass.

One of the first works on the SLIP model is due to Marc Raibert^28^, who showed that SLIP can describe the characteristics of running, trotting or hopping in one leg for bipeds and quadrupeds. Aspects such as stability, dynamics and energy efficiency can be taken into account in this model. Also, Fumiya Iida et al.^29^ showed that walking can be described using the bipedal version of this model. Using the SLIP model, various types of quadruped robots have been development, such as KOLT^7^, Scout II^8^, BigDog^9^, Start1ETH^10^ and the MIT cheetah^30^, which are capable of walking, trotting and galloping at high speeds in different terrains.

However, the SLIP model characterizes the dynamic formulation in a simple way, since it represents the robot’s leg as a point mass and a massless spring that extends towards to the ground. This neglects the inertia of the leg^31^. Hence, this model falls short when it comes to the flight phase of the legs of a galloping robot, i.e. when the leg is not in stance with the ground, since it does not allow generating a force for bending the trunk. In most animals the mass of the leg is very important when performing the galloping movement, especially in quadrupeds with flexible trunks^26^. As mentioned earlier, the mass of the legs helps bending the trunk, allowing it to store and release elastic energy, which allows smoother movements and a more energy efficient gallop^25,27,32^. Therefore, in order to study the effect of the legs’ masses in the dynamics, new models need to be developed. In this paper, we propose a Mass-Mass-Spring (MMS) leg model, as an alternative for quadruped robots with flexible trunk, and demonstrate that considering the mass of the leg in its dynamic modeling, it is possible to control the rotational force at the hip, and therefore induce a bending moment at the end of the trunk in the flight phase.

**Figure 3:**
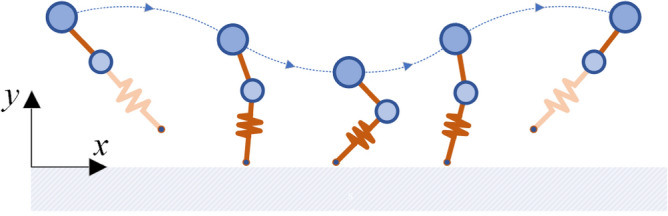
Sequence of the movement of the MMS model. The stance phase in the center of the image and the flight phase is on the sides of the image.

The motion of the leg has two main phases. The *stance phase* represents the system dynamics while it is in contact with the ground and where the spring *k*, which joins the mass *m* of the leg with the ground, acts passively (Fig. 2). Then, the *flight phase* represents the system dynamic during the flight, with the spring resting as the mass *m*. These two phases alternate in time, achieving a continuous movement pattern that causes the system to move along the *x* and *y* coordinates (Fig. 3).

In the following we compare the proposed Mass-Mass-Spring (MMS) leg model with the Spring Loaded Inverted Pendulum (SLIP) model. As can be seen in Fig. 2 (top), the SLIP model is composed of a point mass, *M*, which represents the hip, and a linear spring, *k*, that transmits the reaction forces between the ground and the hip, acting as energy storage during the stance phase. The kinematic and dynamic analyses are explained in the Appendix (see also^33–36^). In addition to the mass of the hip, the MMS model takes into account also the mass of the leg, located at the knee joint, and a spring that establishes the contact between the mass of the leg and the ground, as shown in Fig. 2 (bottom). Thanks to the additional mass *m*, it is possible to model the forces that allow the trunk to bend in the flight phase.

### The Mass-mass-spring model

The dynamic equations that represent the model for stance phase in horizontal and vertical axes are:

1 $$\mathop x\limits^{ \cdot \cdot }_{m} \left( t \right) = \frac{1}{M + m}\left[ {kL_{0} \sin \left( {\alpha - \beta } \right) - kx_{m} \left( t \right) + ML_{hk} \left( {\mathop \alpha \limits^{ \cdot \cdot } \cos \alpha - \dot{\alpha }{\text{asin}}\alpha } \right)} \right]$$

2 $$\mathop x\limits^{ \cdot \cdot }_{M} \left( t \right) = \frac{{kL_{0} \sin \left( {a - \beta } \right) - m\mathop x\limits^{ \cdot \cdot } \left( t \right) - kx_{m} \left( t \right)}}{M}$$

3 $$\mathop y\limits^{ \cdot \cdot }_{m} \left( t \right) = \frac{1}{M + m}\left[ {KL_{0} \cos \left( {\alpha - \beta } \right) - ky_{m} \left( t \right) + \left( {M + m} \right)g + ML_{hk} \left( {\dot{\alpha }cos\alpha + \mathop \alpha \limits^{ \cdot \cdot } \sin \alpha } \right)} \right]$$

4 $$\mathop y\limits^{ \cdot \cdot }_{M} \left( t \right) = \frac{{kL_{0} \cos \left( {\alpha - \beta } \right) - ky_{m} \left( t \right) + Mg + m\left( {g - \mathop y\limits^{ \cdot \cdot }_{m} \left( t \right)} \right)}}{M}$$

where ${\ddot{x}}_{m}$ and ${\ddot{x}}_{M}$ are the horizontal acceleration and ${\ddot{y}}_{m}$ and ${\ddot{y}}_{M}$ are the vertical acceleration of the knee mass, *m*, and hip mass, *M*, respectively, for the stance phase.

The dynamic equations that represent the model for flight phase in horizontal and vertical axes are:

5 $$\mathop x\limits^{ \cdot \cdot }_{m} \left( t \right) = \frac{1}{M + m}\left[ {ML_{hk} \left( {\mathop \alpha \limits^{ \cdot \cdot } \cos \alpha - \dot{\alpha }^{2} \sin \alpha } \right)} \right]$$

6 $$\mathop x\limits^{ \cdot \cdot }_{M} \left( t \right) = - \frac{m}{M + m}\left[ {L_{hk} \left( {\mathop \alpha \limits^{ \cdot \cdot } \cos \alpha - \dot{\alpha }^{2} \sin \alpha } \right)} \right]$$

7 $$\mathop y\limits^{ \cdot \cdot }_{m} \left( t \right) = \frac{1}{M + m}\left[ {\left( {M + m} \right)g + ML_{hk} \left( {\dot{\alpha }\cos \alpha \left( t \right) + \mathop \alpha \limits^{ \cdot \cdot } \sin \alpha } \right)} \right]$$

8 $$\mathop y\limits^{ \cdot \cdot }_{M} \left( t \right) = \frac{{\left( {M - m} \right)g - \left( {\frac{m}{M + m}} \right)\left[ {\left( {M + m} \right)g + ML_{hk} \left( {\dot{\alpha }\cos \alpha + \mathop \alpha \limits^{ \cdot \cdot } \sin \alpha } \right)} \right]}}{M}$$

where ${\ddot{x}}_{m}$ and ${\ddot{x}}_{M}$ are the horizontal acceleration and ${\ddot{y}}_{m}$ and ${\ddot{y}}_{M}$ are the vertical acceleration of the knee mass, *m*, and hip mass, *M*, respectively, for the flight phase.

## Methods

In order to assess the impact that the mass of the leg has on the spine of a quadruped legged robot, we considered the two different types of gallop, the so-called “rotary” and “transverse” gallop^26^.

The rotary gallop is employed, e.g., by the cheetah^26,37^, while the transverse gallop is employed by the horse^38^. In both modes it can be observed how the mass of the leg affects the bending of the trunk. To compare these kinds of gallop we analyzed six species of animals, three for each type gallops: cheeta, greyhound and lynx for rotatory gallop, and horse, antelope and alpaca for transverse gallop^39^.

Figure 1 shows the differences between the two gallops. The rotatory gallop starts with a footfall of one of the forelimbs, to later support with the contralateral forelimb. After that, the flight phase starts, where the legs are completely below itself, and then continue a footfall with the ipsilateral hindlimb and support with the contralateral hindlimb, and then carry out another flight phase where the legs are extended. In contrast, the transverse gallop starts with one footfall of the hindlimbs and later to support with the contralateral hindlimb, following by the contralateral support of the ipsilateral forelimb and later a footfall of the contralateral forelimb, and then carry out flight phase, where the legs are completely below the body^26,39^.

**Figure 4:**
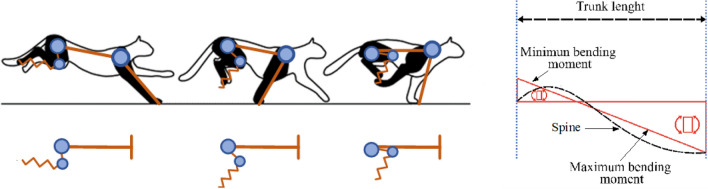
Left: Simplified diagram of a beam with joints for the study of the forces exerted on the trunk. Right: analytical diagram of the minimum bending moment, at the free end, and the maximum bending moment, at the fixed end.

Figure 4 (bottom-left), shows the diagram of the simulation setup for measuring the energy that can be stored in the trunk thanks to the mass of the leg, located in the knee joint. The leg is attached at one end of a beam, while the other end of the beam was fixed. The aim of this setup is to measure the forces that are exerted at the ends of the beam representing the trunk of the robot.

**Figure 5:**
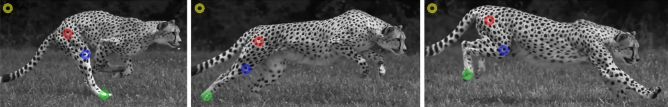
Sequence example of the data acquired by Tracker using the video^3^, red circle follows the hip, the blue circle follows the knee and green circle follows the foot of the cheetah. The yellow circle is a reference to correct the acquired data.

In order to compare the MMS and the SLIP model, the following Key Performance Indicators (KPIs) were used: stride length and hopping height, which are used to evaluate the performance of quadruped robots^30,31^, and normal effort, tangential stress, minimum bending moment, maximum bending moment, and bending moment difference, which are used to know the stresses in the beam^40,41^. Here, the most important indicators are the minimum bending moment, maximum bending moment (see Fig. 5, right), that induce the bending at the two endings of the spine.

**Figure 6:**
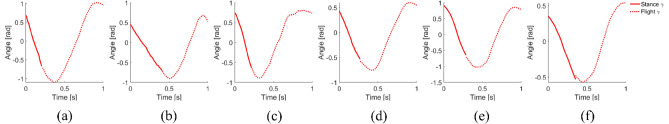
Input γ angles for the simulation of SLIP model for stance and flight phases, calculated from the acquired movement for the (**a**) cheetah, (**b**) greyhound, (**c**) lynx, (**d**) horse, (**e**) antelope and (**f**) alpaca.

**Figure 7:**
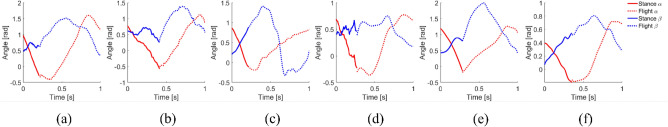
Inputs α and ß angles for the simulation of the MMS model for stance and flight phases, calculated from the acquired movement for the (**a**) cheetah, (**b**) greyhound, (**c**) lynx, (**d**) horse, (**e**) antelope and (**f**) alpaca.

For the purpose of comparison, the same physical dimensions have been adopted for the two motion patterns, (see Table 1). The physical properties of the hind leg of a cheetah Acinonyx Jubatuss, obtained from^42^, were taken into account. In order to assess the effectiveness of the two models, we carried out simulations of a galloping cheetah, greyhound and lynx (rotatory gallop) and horse, antelope and alpaca (transverse gallop), using the kinematics and dynamics equations of the models. To acquire the movements described by the hip, knee and foot we analyzed footage of the running sequence of the two animals (”Maverick Galopp”, 2012, https://youtu.be/iWGKOHeSpE0; “The Science of a Cheetah’s Speed”—National Geographic and the Cincinnati Zoo, 2013, https://youtu.be/icFMTB0Pi0g) using the Tracker software (Tracker is a video analysis and modeling tool built on the Open Source Physics (OSP),https://physlets.org/tracker/) for the stance and flight phases (Fig. 5). With the data acquired, the angles α, ß and γ are calculated. These will be the inputs for each of the simulation models. Figure 6 depicts the gamma angles for SLIP model, and Fig. 7 shows the α and ß for MMS model. To perform the simulation, and to visualize of the movements, the Robotics toolbox system of MathWorks was used.

**Table 1:** Physical parameters of the models used.

Parameter	Expression	Value
Femur length	*L*_*hk*_	0.294 m
Tibia length	*L*_*kf*_	0.298 m
Spring length	*L*	0.298 m
Total length	*L*_*total*_	0.592 m
Hip mass in MMS model	*M*	7.296 kg
Knee mass in MMS model	*m*	0.454 kg
Total mass in SLIP model	*M*_*total*_	7.75 kg
Backbone length	*L*_*col*_	0.7 m
Spring constant	*k*	2000 N/m
Initial y axis velocity	*vy*	− 1.5 m/s
Initial x axis velocity	*vx*	0 m/s

## Results

**Figure 8:**
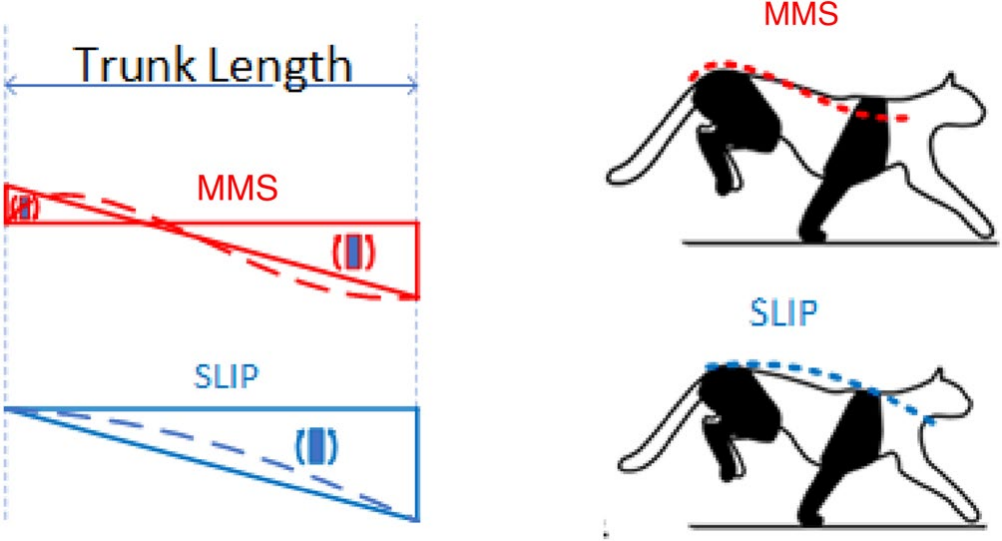
Scheme of the deformation for the bending moments of the MMS leg model and SLIP leg model in flight phase.

Tables 2, 3 and 4 report the results of the cheetah, greyhound and lynx simulations. Although the stride length is bigger in the MMS model and the hopping is slightly bigger in SLIP model, it is important to note the difference in the minimum and maximum bending moments of the two models. In particular, a *minimum bending moment* is obtained with the MMS mode, while this is null in the SLIP model. Hence, the MMS model is capable of generating a more accurate bending of the trunk (Fig. 8). This is because it is the *minimum bending moment* that causes a bending of the trunk, similar to one of the cheetah galloping. The SLIP model only allows to obtain a buckling in the spine.

**Table 2:** Key performance indicators cheetah trajectory.

Parameter	MMS	SLIP
Stride length	2.152 m	1.4834 m
Hopping height	0.31969 m	0.35304 m
Normal effort	16.1752 N	0 N
Tangential stress	− 76.0275 N	− 76.0275 N
Minimum bending moment	3.8819 Nm	0 Nm
Maximum bending moment	− 49.3374 Nm	− 53.22 Nm
Bending difference	53.22 Nm	53.22 Nm

**Table 3:** Key performance indicators greyhound trajectory.

Parameter	MMS	SLIP
Stride length	2.1254 m	0.945 m
Hopping height	0.22992 m	0.20422 m
Normal effort	25.7051 N	0 N
Tangential stress	− 76.0275 N	− 76.0275 N
Minimum bending moment	4.8255 Nm	0 Nm
Maximum bending moment	− 48.3937 Nm	− 53.2193 Nm
Bending difference difference	53.2192 Nm	53.2193 Nm

**Table 4:** Key performance indicators lynx trajectory.

*Parameter*	*MMS*	*SLIP*
Stride length	1.4197 m	0.84341 m
Hopping height	0.2901 m	0.31649 m
Normal effort	17.2926 N	0 N
Tangential stress	− 76.03 N	− 76.03 N
Minimum bending moment	3.1968 Nm	0 Nm
Maximum bending moment	− 50.0225 Nm	− 53.2192 Nm
Bending difference difference	53.2192 Nm	53.2192 Nm

Similar results are obtained in the horse, antelope and alpaca, transverse gallop, reported in Tables 5, 6 and 7. As for the cheetah, in the trajectory of the horse it can be noticed an increase in the minimum bending moment and a decrease in the maximum bending moment for the MMS model, compared to the SLIP model. Again, this is due to the mass of the leg, which induces a rotational force in the hip, which in turn, causes a bending moment to be generated in the spine.

**Table 5:** Key performance indicators horse trajectory.

Parameter	MMS	SLIP
Stride length	1.44 m	0.72886 m
Hopping height	0.20644 m	0.19122 m
Normal effort	10.9171 N	0 N
Tangential stress	− 76.027 N	− 76.027 N
Minimum bending moment	2.1319 Nm	0 Nm
Maximum bending moment	− 51.0873 Nm	− 53.21 Nm
Bending difference difference	53.2192 Nm	53.21 Nm

**Table 6:** Key performance indicators antelope trajectory.

Parameter	MMS	SLIP
Stride length	1.5651 m	1.0339 m
Hopping height	0.38338 m	0.45287 m
Normal effort	4.623 N	0 N
Tangential stress	− 76.03 N	− 76.03 N
Minimum bending moment	1.7279 Nm	0 Nm
Maximum bending moment	− 51.4913 Nm	− 53.2192 Nm
Bending difference difference	53.2192 Nm	53.2192 Nm

**Table 7:** Key performance indicators alpaca trajectory.

Parameter	MMS	SLIP
Stride length	0.62446 m	0.48691 m
Hopping height	0.15274 m	0.16222 m
Normal effort	4.9778 N	0 N
Tangential stress	− 76.027 N	− 76.027 N
Minimum bending moment	1.5495 Nm	0 Nm
Maximum bending moment	− 51.6698 Nm	− 53. 2193 Nm
Bending difference difference	53.2193 Nm	53. 21,923 Nm

It can be noted that for the “bending difference” parameter (the difference between minimum and maximum bending,) the results are similar in the two models, but the SLIP model produces no “minimum bending”. This is because in the MMS model the mass of the leg is taken into account (about 6% of the total mass), generating the bending moment at the end of the spine. In MMS A lower maximum bending at the fixed end of spine, is generated. Note that the total mass is equal in the two models (*M* + *m* in MMS, *M*_*total*_ in SLIP).

The tangential stress parameter is useful because it helps to know what force stretches the spine in galloping. This is similar for the two models, despite the masses are distributed between hip and knee in the MMS model while it is concentrated at the hip in the SLIP model.

**Figure 9:**
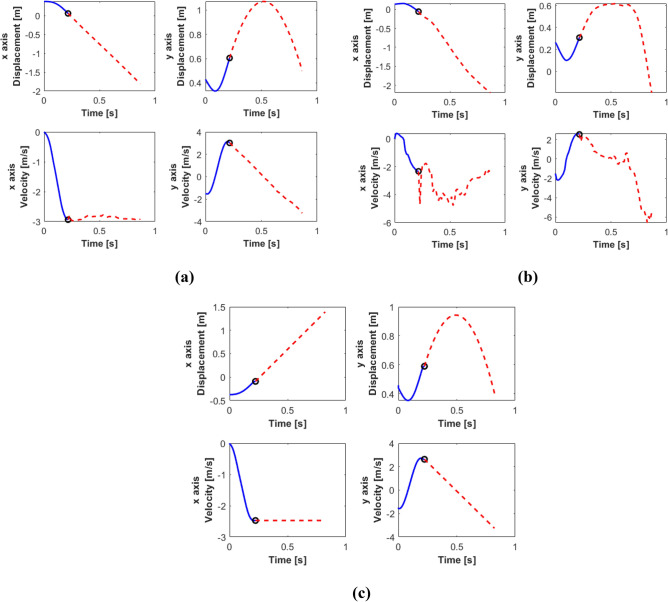
*×* and *y* position and velocity of the (**a**) hip and the (**b**) knee of the MMS leg model and the (**c**) hip of the SLIP leg model in the galloping movement of the cheetah. Solid line: stance phase; dashed line: flight phase.

**Figure 10:**
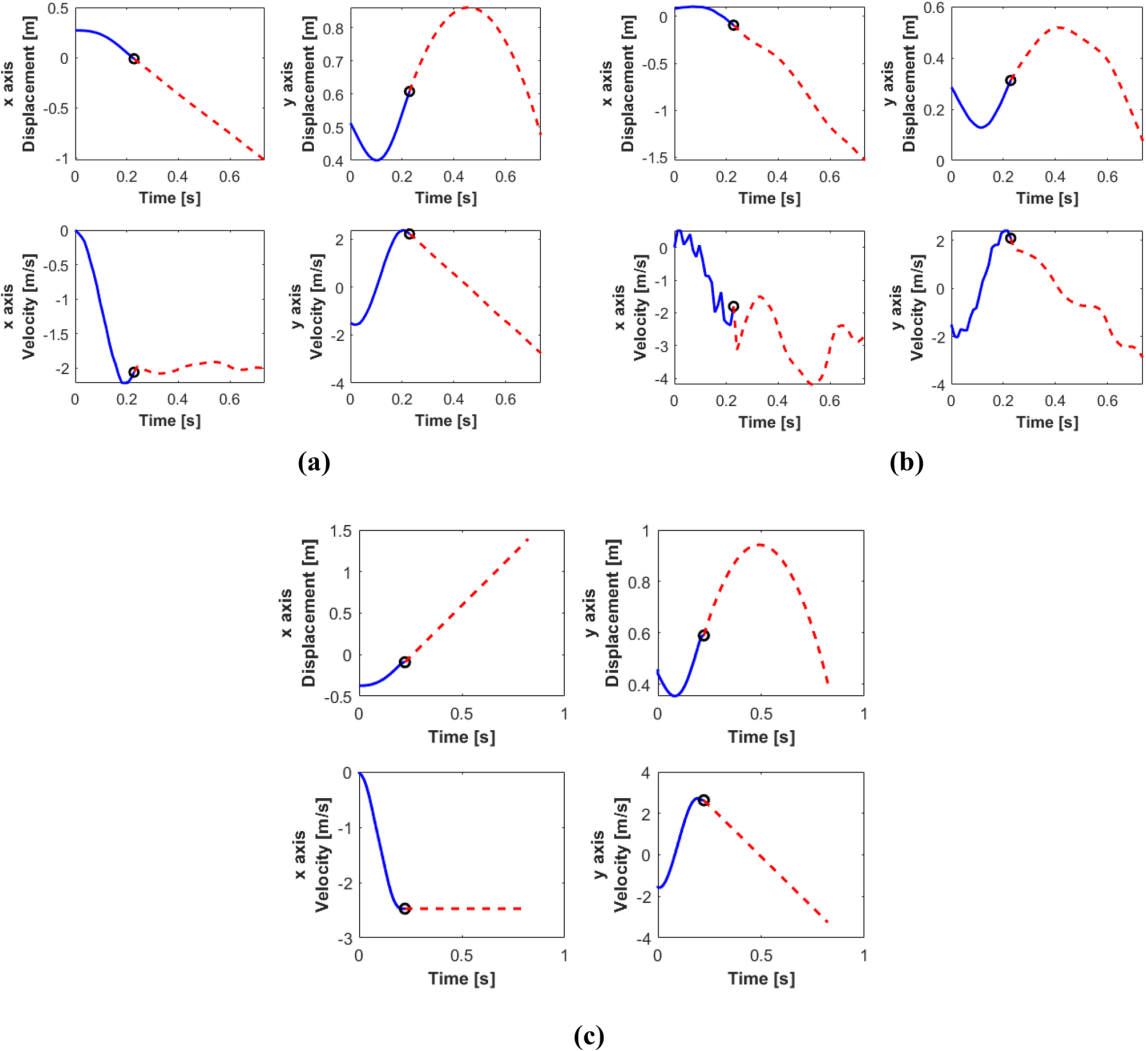
*×* and *y* position and velocity of the (**a**) hip and the (**b**) knee of the MMS leg model and the (**c**) hip of the SLIP leg model in the galloping movement of the horse. Solid line: stance phase; dashed line: flight phase.

Figures 9 and 10 show the details of the displacement and velocity of the hip and knee resulting from the simulation of stance phase (solid line) and flight phase (dashed line) for the cheetah and the horse, considering the parameters of Table 1. We choose these two anymals as representative of their respective groups, because < < *the transverse gallop is epitomized by the horse and the rotary gallop by the cheetah* > > ^26^.

Figures 9a,c and 10a,c depict the movement of the hip, in MMS and SLIP models, describes a parabolic movement in the flight phase, since the heaviest mass is at the hip drives the trajectory of the leg in the flight phase. Similarly, as can be seen in Figs. 9b and 10b, there are abrupt changes in velocities, both in stance phase and flight phase. These are due to the mass in the knee which allows to obtain a moment of force at the end of the spine that helps to accumulate energy during flight phase, and releases energy during the stance phase.

## Discussion

The purpose of our current work is to understand and model the mechanics of bending of the trunk in quadrupeds with flexible trunk, with the aim of reproducing the mechanism of storing/releasing energy during galloping. This is a key feature for improving the performance of quadruped robots. Additionally, a flexible trunk helps a smooth deceleration and a fast acceleration of the different parts of the body involved during running, as well as it reduces shocks in the mechanical structure at the benefit of lighter structures and smoother movements.

We have demonstrated how using the modeling the gallop of a quadruped robot with flexible trunk by using the proposed MMS leg model produces better results with respect to the commonly used SLIP leg model. This model allows a moment of force to be generated at the center of mass of the hip when performing the galloping movement, called minimum bending moment. This is because in the MMS model the mass of the leg is taken into account (about 6% of the total mass), generating the bending moment at the end of the spine. Note that the total mass is equal in the two models (*M* + *m* in MMS, *M*_*total*_ in SLIP). Due to its mathematical formulation, the SLIP model cannot reproduce the bending moment at the free end of the spine. This is precisely the reason we developed the MMS model.

Even if the purpose of the paper is not a comparative analysis of biological data, but to assess the goodness of the proposed mathematical model, some conclusion from the experiments can be drawn, keeping in mind that the small number of animals analyzed does not allow an in-depth statistical analysis.

Comparing the KPIs of the six animals analyzed, it can be seen that in general, the animals that employ rotary gallop have a higher bending moment, while a transversal gallop produces a higher maximum bending moment (Table 8). The transversal gait begins positioning the hind legs on the ground and the front legs in the flight phase, generating a rotational force that allows generating a maximum bending moment higher than the minimum bending moment on its hind limb. On the contrary, the rotary gait begins positioning front legs on the ground and the hind legs in the flight phase, generating a high rotational force in its rear part and consequently a bigger value of the minimum bending moment, allowing a bigger flexion in the trunk and therefore a higher energy storage, which allows more powerful gait when “launching” its forequarters in the beginning of the aerial phase. Therefore, the animals that perform a rotatory gallop, seem to be better at storing and releasing energy. This may explain the higher velocity of the animals employing rotary gallop compared to the animals that employ transversal gallop.

**Table 8:** Summary of the KPIs.

Parameter	Rotary		Transversal	
	Average	St. dev	Average	St. dev
Stride length	1.90	0.42	1,21	0,51
Hopping height	0.28	0.05	0.25	0.12
Normal effort	19.72	5.21	6.84	3.54
Tangential stress	− 76.03	0.00	− 76.03	0.00
Minimum bending moment	3.97	0.82	1.80	0.30
Maximum bending moment	− 49.25	0.82	− 51.42	0.30
Bending difference	53.22	0.00	53.22	0.00

Indeed, as Biancardi Minetti, point out after the analysis of 89 mammalian species^39^, even if more than 80% of the species they analyzed use only one kind of gallop at any speed, the other 20% show a preference for transverse canter or gallop at slow speed, and rotary gallop at higher travel speeds. Their results also indicated that < < *slower and larger mammals, with relatively longer and thicker limbs, predominantly employ transverse gallop. In contrast, lighter and faster mammals, which have relatively shorter legs and longer body, were more likely to use rotary gallop* > > . We believe this is in agreement with the results of our simulations.

This work has focused on gallop, because this is the running sequence where the effect of the bending of the trunk and the accumulation/release of energy is most important. Future work might be devoted to the analysis of different running sequences (e.g., trot) in order to assess the relative behavior of the SLIP and MMS models (and possibly others) and to understand the benefits of implementing this kind of locomotion in quadrupedal robots.

## Supplementary Information

Supplementary Information.

## Data Availability

The datasets generated and analyzed during the current study are available in the Simulation_Data repository (https://github.com/eaparra01/Simulation_Data). The code of the two models is available at https://github.com/chechugador/MMR.
