# Supplementary material for: Comparison of leg dynamic models for quadrupedal robots with compliant backbone
Source: Sci Rep. 2022 Aug 26;12:14579. doi: 10.1038/s41598-022-18536-7 (PMC9418320; doi:10.1038/s41598-022-18536-7)
Supplement: Supplementary file 1 — Supplementary Information. [file 41598_2022_18536_MOESM1_ESM.docx]

Comparison of Leg Dynamic Models for Quadrupedal Robots with Compliant Backbone

E. A. Parra Ricaurte⸸, J. Pareja⸸, S. Dominguez⸸, C. Rossi*⸸

# Appendix.

In this section, the kinematic and dynamic formulation for the MMS model and SLIP model is explained.

## Kinematic and dynamic of the MMS Model

The kinematic study is divided into the two phases described above (see also Figure 2). During the stance and flight phases, the following trigonometric functions are obtained for the hip and knee joint:

|  | $x_{M}\left( t \right)=x_{m}\left( t \right)-L_{hk}\sin\alpha\left( t \right)$ | (1) |
| --- | --- | --- |
|  | $y_{M}\left( t \right)=y_{m}\left( t \right)+L_{hk}\cos\alpha\left( t \right)$ | (2) |
|  | $L\left( t \right)=\frac{x_{m}\left( t \right)}{\sin\left( \alpha\left( t \right)-\beta\left( t \right) \right)}=\frac{y_{m}\left( t \right)}{\cos\left( \alpha\left( t \right)-\beta\left( t \right) \right)}$ | (3) |

Where *L(t)* is the spring length with its stiffness constant, *k*, *L_hk_* is the bar length that is between the hip and knee (see Fig. 2, bottom). Similarly, *x_M_*, *y_M_* are the position of the hip mass and *x_m_*, *y_m_* are the position of the leg mass, that it is in the joint knee position, *α* is the angle of the bar, *L_hk_*, with respect to the vertical axis in the hip mass position and *β* is the angle of the spring rotates with respect to the bar, *L_hk_*, in the knee position.

For the flight phase, equation 3 is not considered since the spring does not change its effective length because no force is exerted on it. Then, the dynamic analysis in stance and flight phases was obtained using Newton’s second law of motion.

For the stance phase, the horizontal axis equation that defines the dynamic behavior is:

|  | $M\ddot{x}_{M}\left( t \right)+m\ddot{x}\left( t \right)=k\left( L_{0}-L\left( t \right) \right)\sin\left( a\left( t \right)-\beta\left( t \right) \right)$ | (4) |
| --- | --- | --- |

By evaluating the right side of equation (4), including the second term of equation (3) for *x_m_*, we obtain an expression that does not depend on the value of the length of the spring at each instant.

|  | $M\ddot{x}_{M}\left( t \right)+m\ddot{x}\left( t \right)=kL_{0}\sin\left( a\left( t \right)-\beta\left( t \right) \right)-kx_{m}(t)$ | (5) |
| --- | --- | --- |

Now, deriving the equation (1) twice, we have the velocity, $\dot{x}_{M}(t)$, and the acceleration, $\ddot{x}_{M}(t)$, and including the acceleration result into the equation (5), we obtain:

|  | $\left( M+m \right)\ddot{x}_{m}(t)+ML_{hk}\left( \dot{\alpha}^{2}\sin\alpha\left( t \right)-\ddot{\alpha}\cos\alpha\left( t \right) \right)=kL_{0}\sin\left( \alpha\left( t \right)+\beta\left( t \right) \right)-kx_{m}(t)$ | (6) |
| --- | --- | --- |

So we obtain:

|  | $\ddot{x}_{m}(t)=\frac{1}{M+m}\left[ kL_{0}\sin\left( \alpha\left( t \right)-\beta\left( t \right) \right)-kx_{m}(t)+ML_{hk}\left( \ddot{\alpha}\cos\alpha-\dot{\alpha}\mathrm{asin} \alpha\right) \right]$ | (7) |
| --- | --- | --- |

This equation represents the dynamic behavior of the model in its horizontal axis and depends on the angular velocity and acceleration, as well as on the configuration adopted for the turns of each joint.

For the stance phase for the vertical axis, the balance of forces is proposed for the component considering the action of gravity on each of the point masses. The equation that defines the dynamic behavior is:

|  | $M\ddot{y}_{M}(t)+m\ddot{y}_{m}(t)=k\left( L_{0}-L\left( t \right) \right)\cos\left( \alpha\left( t \right)-\beta(t) \right)+Mg+mg$ | (8) |
| --- | --- | --- |

Evaluating the right side of equation (8), including the third term of equation (3) for *y_m_*, we obtain an expression that does not depend on the value of the length of the spring at each instant.

|  | $M\ddot{y}_{M}(t)+m\ddot{y}_{m}\left( t \right)=kL_{0}\cos\left( \alpha\left( t \right)-\beta\left( t \right) \right)-ky_{m}(t)+Mg+mg$ | (9) |
| --- | --- | --- |

Later, deriving the equation (2) twice we obtain the components of velocity, $\dot{y}_{M}$, and acceleration, $\ddot{y}_{M}$, and including the acceleration result into the equation (9), we obtain:

|  | $\ddot{y}_{m}(t)=\frac{1}{M+m}\left[ KL_{0}\cos\left( \alpha\left( t \right)-\beta\left( t \right) \right)-ky_{m}(t)+\left( M+m \right)g+ML_{hk}\left( \dot{\alpha} cos\alpha\left( t \right)+\ddot{\alpha}\sin\alpha\left( t \right) \right) \right]$ | (10) |
| --- | --- | --- |

As for the horizontal axis, this equation represents the dynamic behavior of the model in its vertical axis, depending on the angular velocity and acceleration, and therefore, the configuration adopted by the model according to the turns of each joint. Equations (7) and (10) define the dynamic behavior of the model in the stance phase.

For the flight phase, the same methodology was followed. When performing the equilibrium of forces on the horizontal axis, taking into account that the spring force, friction with the air and other aerodynamic aspects are neglected. We obtain:

|  | $M\ddot{x}_{M}\left( t \right)+m\ddot{x}_{m}\left( t \right)=0$ | (11) |
| --- | --- | --- |

Taking equation (1) and performing the two derivatives obtaining $\dot{x}_{M}$ and $\ddot{x}_{M}$, the components of velocity and acceleration respectively, and including the acceleration result in equation (11) and then clearing for $\ddot{x}_{m}$, we obtain:

|  | $\ddot{x}_{m}(t)=\frac{1}{M+m}\left[ ML_{hk}\left( \ddot{\alpha}\cos\alpha-\dot{\alpha}^{2}\sin\alpha\right) \right]$ | (12) |
| --- | --- | --- |

For the equilibrium of forces, on the vertical axis, the action of gravity appears.

|  | $M\ddot{y}_{M}\left( t \right)+m\ddot{y}_{m}\left( t \right)=Mg+mg$ | (13) |
| --- | --- | --- |

Taking equation (2) and performing the two derivative we obtain $\dot{y}_{M}$ and $\ddot{y}_{M}$, the components of velocity and acceleration respectively. Then, including the acceleration result in the equation (13), and then clearing for $\ddot{y}_{m}$ we obtain:

|  | $\ddot{y}_{m}(t)=\frac{1}{M+m}\left[ \left( M+m \right)g+ML_{hk} \left( \dot{\alpha}\cos\alpha\left( t \right)+\ddot{\alpha}\sin\alpha\left( t \right) \right) \right]$ | (14) |
| --- | --- | --- |

Equations (12) and (14) define the dynamic behavior of the model in the flight phase. These functions depend on the angular velocity and acceleration, gravity and therefore, the configuration adopted by the model according to the turns of each joint.

## Kinematic and dynamic of SLIP Model

For our research, the SLIP model was used for comparison, since it has been intensively used both to study the
behavior of the most common running movement sequences in the animal kingdom, and to serve as an initial template in the design and development of leg-based locomotion solutions for mobile robotics. As can be seen in Figure 2 (top), the model is composed of a point mass, *M*, which represents the center of mass, (*CoM*), a linear spring, *k*, the one that transmits the reaction forces between the ground and the mass, where this acting as energy storage during the stance phase as well. Equally the MMS model, the kinematic and dynamic of the SLIP model are divided into stance phase and flight phase. By computing the equilibrium forces for the stance phase of the model, for the horizontal and vertical axes, the following equations are obtained:

|  | ${M\ddot{x}}_{M}(t)=kx_{M}(t)\left( \frac{l_{0}-l(t)}{l} \right)$ | (15) |
| --- | --- | --- |

|  | ${M\ddot{y}}_{M}(t)=ky_{M}(t)\left( \frac{l_{0}-l(t)}{l(t)} \right)-Mg$ | (16) |
| --- | --- | --- |

It can be observed that both the spring and gravity influence on dynamics of the model, where *k* is the stiffness constant, *l* is the length change and $l_{0}$ is the length at rest state of the spring. By defining the angle γ that the spring forms with respect to the vertical axis, and clearing $\ddot{x}_{M}$ and $\ddot{y}_{M}$, the dynamic equations in the stance phase are obtained for the horizontal and vertical axes:

|  | $\ddot{x}_{M}(t)=\frac{kx_{M}\sin\gamma(t)}{M}$ | (17) |
| --- | --- | --- |

|  | $\ddot{y}_{M}(t)=\frac{kx_{M}\cos\gamma\left( t \right)-Mg}{M}$ | (18) |
| --- | --- | --- |
|  |  |  |

During the flight phase, the equations are determined by the parabolic motion, since by neglecting the spring
force, the friction forces of the air and other aerodynamical considerations, and applying Newton’s second
law, we obtain:

|  | ${M\ddot{x}}_{M}(t)=0$ | (18) |
| --- | --- | --- |

|  | ${M\ddot{y}}_{M}(t)=- Mg$ | (19) |
| --- | --- | --- |

And obtain the temporal evolution of the coordinates in $x_{M}$ and $y_{M}$ of the (*CoM*) solving the previous equation.

|  | $x_{M}(t)= x_{0}+\dot{x}_{M}(t)$ | (20) |
| --- | --- | --- |

|  | $y_{M}(t)= y_{0}+\dot{y}_{M}\left( t \right)-\frac{1}{2}gt^{2}$ | (21) |
| --- | --- | --- |

For more details about this model, we refer the reader to [32-33].
